# Peer feedback decreases impulsive choice in adolescents with and without attention‐deficit/hyperactivity disorder

**DOI:** 10.1002/jcv2.12065

**Published:** 2022-02-25

**Authors:** Jorien van Hoorn, Erik de Water, Tycho J. Dekkers, Yehuda Pollak, Arne Popma, Brenda R. J. Jansen, Hilde M. Huizenga, Anna C. K. van Duijvenvoorde

**Affiliations:** ^1^ Institute of Psychology, Department of Developmental and Educational Psychology Leiden University Leiden The Netherlands; ^2^ Leiden Institute for Brain and Cognition Leiden The Netherlands; ^3^ Levvel Academic Center for Child‐ and Adolescent Psychiatry Amsterdam The Netherlands; ^4^ Fraser Minneapolis Minnesota USA; ^5^ Department of Psychology University of Amsterdam Amsterdam The Netherlands; ^6^ Department of Child‐ and Adolescent Psychiatry University Medical Center Groningen Groningen The Netherlands; ^7^ Department of Child‐ and Adolescent Psychiatry Amsterdam University Medical Centers Amsterdam The Netherlands; ^8^ Seymour Fox School of Education Hebrew University of Jerusalem Jerusalem Israel; ^9^ Amsterdam Brain and Cognition Center Amsterdam The Netherlands; ^10^ Research Priority Area Yield Amsterdam The Netherlands

**Keywords:** ADHD, adolescence, impulsivity, peer feedback, social context

## Abstract

**Background:**

Impulsivity is a core feature of attention‐deficit/hyperactivity disorder (ADHD). Previous work using the delay discounting task to assess impulsivity reveals that adolescents with ADHD tend to prefer a smaller‐immediate reward over a larger‐delayed reward, and this relates to problematic choices in daily life. To gain a better understanding of daily decision‐making in adolescence, it is important to examine the social context, as peers have a major influence on decisions. Peer influence often has a negative connotation, but also provides an opportunity to promote positive outcomes. To date, it is unclear if peers affect impulsive decision‐making in adolescents with ADHD, for better or for worse.

**Methods:**

The aim of this preregistered study was to examine the effect of peer feedback on impulsive choice in male adolescents with and without ADHD (ages 13–23; *N* = 113). We utilized an adapted delay discounting task that was administered alone, in a social condition, and alone again. In the social condition, adolescents received either (between‐subjects) manipulated impulsive or non‐impulsive peer feedback. Impulsive peer feedback consisted of likes for choosing the smaller immediate reward, whereas non‐impulsive peers endorsed choosing the larger delayed reward.

**Results:**

Preregistered analyses showed that non‐impulsive peer feedback resulted in decreased impulsive choice, whereas impulsive peer feedback did not alter decision‐making in adolescents with and without ADHD. Explorative analyses of inattention and hyperactivity‐impulsivity symptoms in the total sample, irrespective of diagnosis, showed that lower hyperactivity–impulsivity and more inattention symptoms were associated with increased susceptibility to non‐impulsive peer feedback.

**Conclusions:**

Together, these findings indicate that peers may provide an opportunity to decrease impulsivity and emphasize individual differences in susceptibility to non‐impulsive peer feedback related to inattention and hyperactivity–impulsivity. Therefore, peer feedback may be a promising component in behavioral peer‐supported interventions in adolescents with ADHD.

**Key points**

- Impulsive choice in adolescents with ADHD relates to problematic choices in daily life and can be measured using the delay discounting task.
- Given the social reorientation of adolescence, peer influence may provide an opportunity to impact impulsive choice in adolescence, for better or for worse.
- Our results revealed that non‐impulsive peer feedback results in decreased impulsive choice.
- Across the total sample, irrespective of diagnosis, more inattention symptoms were associated with increased susceptibility to non‐impulsive peer feedback, whereas more hyperactivity–impulsivity symptoms were related to reduced susceptibility to non‐impulsive peer feedback.
- Our findings are relevant to a basic understanding of social processes in adolescents with ADHD, and suggest that peer influence can be a promising component in interventions to decrease impulsive behaviors in ADHD.

Impulsivity is a core feature of attention‐deficit/hyperactivity disorder (ADHD) and has frequently been measured using delay discounting tasks in children and adolescents with ADHD (Barkley, 1997; Jackson & MacKillop, 2016; Marx et al., 2021; Patros et al., 2016). Delay discounting refers to the decrease in the subjective value of a reward as the delay preceding its receipt increases (Critchfield & Kollins, 2001). Several meta‐analyses have shown that children and adolescents with ADHD, relative to those without ADHD, show a greater preference for smaller immediate rewards over larger delayed rewards in delay discounting tasks (Jackson & MacKillop, 2016; Marx et al., 2021; Patros et al., 2016). The preference for smaller, immediate rewards has been related to risk‐taking and problematic choices in everyday life, such as substance use and smoking (Amlung et al., 2016; Audrain‐McGovern et al., 2009; Jackson & MacKillop, 2016). This empirical work on impulsivity resonates with the dual path‐way theory of ADHD (Sonuga‐Barke, 2002, 2003), which proposes that ADHD may be characterized by differential reward processing (Luman et al., 2005; Marx et al., 2021; Patros et al., 2016), and impaired executive functions such as working memory, inhibition and planning (Martinussen et al., 2005; Willcutt et al., 2005).

Given the serious consequences of impulsive and risky behaviors for the individual and society (Faraone et al., 2021), it is important to investigate possibilities to reduce impulsivity in ADHD, particularly during adolescence as this development period is characterized by a peak in impulsive and risky decision‐making (Dekkers et al., 2022; Rosenbaum & Hartley, 2019). To date, early work in impulsive preschoolers has suggested that training reward immediacy has promising effects on impulsivity through an extensive shaping procedure, but recent work is lacking and sample sizes are small (Schweitzer & Sulzer‐Azaroff, 1988; reviewed in Rutledge et al., 2012). At the same time, research has shown that training of cognitive and executive functions has limited effects in individuals with ADHD (Cortese et al., 2015; Rapport et al., 2013). Taken together, new avenues need to be explored to enhance and supplement the previous work done in this area.

During adolescence, a characteristic social reorientation takes place during which peers' opinions become highly salient (Andrews et al., 2021). As such, one key factor that may affect adolescents' impulsivity is the context of peers (Albert et al., 2013). Experimental work illustrates that adolescents prefer smaller‐immediate rewards over larger‐delayed rewards, when they make decisions in the presence of peers (O’Brien et al., 2011; Weigard et al., 2014). Moreover, if young adults observe peer responses that favor the smaller‐immediate reward in a delay discounting task, their own decisions also become more impulsive (Gilman et al., 2014). A neuroimaging study examined the effects of peer presence in the brain, and demonstrated heightened activation of the reward circuitry (i.e., ventral striatum; Chein et al., 2011). Taken together, this suggests that peers may increase the perceived value of (immediate) rewards (Chein et al., 2011; Weigard et al., 2014), and thus may impact the reward pathway of the brain which is thought to function differently in ADHD (Sonuga‐Barke, 2002, 2003; also see Rubia, 2018).

Peer influence may not only lead to more impulsive choices, but could also reduce such behaviors through social learning (Akers, 2011; Bandura, 2001). Social learning theory describes a process of observing and imitating behaviors from others around us, especially close others such as parents or friends (Bandura, 2001). More specifically, peer influence is thought to work through internalizing social norms from the peer context, which designate which behaviors will be accepted and approved by peers (McDonald & Crandall, 2015) and may provide social status. While even the mere presence of peers increases the likelihood of risky driving in adolescents (implicit social norms), the impact of peer feedback (explicit social norms) on decision‐making is generally even larger (Chein et al., 2011; Munoz Centifanti et al., 2016). Currently, it is unknown if peer feedback could potentially sway adolescents away from impulsive decisions. Such positive effects of peer feedback have already been shown in the domain of prosocial behavior (Van Hoorn, van Dijk, Meuwese, et al., 2016). If non‐impulsive peer feedback similarly affects impulsive choice, this could be an important component for intervention in adolescents with ADHD, such as behavioral peer interventions (Evans et al., 2018).

Adolescents with ADHD may be especially susceptible to peer feedback as they experience more peer rejection, have difficulties managing peer relations and associate with deviant peer groups more often (Bagwell et al., 2001; Ferguson, 2000; Nijmeijer et al., 2008). Those with the predominantly inattentive presentation of ADHD are more impaired in assertiveness, while the combined presentation is associated with a deficiency in self‐control (Solanto et al., 2009), and each results in social problems (McQuade, 2020). Therefore, conforming to peers might be a means to be accepted in a group and perhaps to gain status (Brechwald & Prinstein, 2011; Cialdini & Goldstein, 2004). Interestingly, a recent study showed that adolescents with ADHD were equally sensitive to influence from a single risky peer as typically developing adolescents (Dekkers et al., 2020). The current work builds on these findings by examining whether peer feedback from a *group* of peers similarly affects *impulsive* decision‐making.

## PRESENT STUDY

Given the potential of peers to influence decision‐making in adolescence, and the clinical relevance in behavioral peer interventions, the main goal of this preregistered study was to investigate to what extent adolescent males (ages 13–23) with and without ADHD are influenced by peer feedback on impulsive choice and how peer effects are shaped by impulsive and non‐impulsive social norms. We exploratively tested whether differences in inattention and hyperactivity–impulsivity symptoms (irrespective of diagnosis) may play a role in individual differences in susceptibility to peer feedback. We focused on this age range because peer influence is highly salient during adolescence (up to 24 years, Sawyer et al., 2018), and only slowly decreases in young adulthood (Andrews et al., 2021; Knoll et al., 2015). These theoretical considerations, together with practical considerations considering sex,^1^ led to the inclusion of a male‐only sample of 13‐ to 23‐year‐old.

To examine peer influence on impulsive choice we adapted a delay discounting task, which was completed alone and in a between‐subjects social condition. After a few rounds of decision‐making alone, adolescents received manipulated peer feedback on their decisions, which consisted of ‘likes’ or thumbs up from five peers (cf. Van Hoorn, Van Dijk, Guroglu et al., 2016). These supposed peers provided either impulsive or non‐impulsive peer feedback. In the impulsive peer feedback condition, choosing the smaller immediate reward resulted in getting the majority of likes, whereas this was the exact opposite in the non‐impulsive peer feedback condition. After a few rounds with peer feedback, adolescents continued to play alone again. This allows us to examine a potential carry‐over effect, that is, whether social norms from peers are subsequently implemented in decision‐making, when peers are no longer providing feedback.

We expect that adolescents with and without ADHD are sensitive to peer feedback on delay‐discounting decisions. In typically developing adolescents, the mere presence of peers or observation of a peers' impulsive delay discounting behavior leads to more impulsive choices, presumably due to effects on perceived value of reward (Gilman et al., 2014; O’Brien et al., 2011; Weigard et al., 2014). Previous work in the prosocial domain illustrated strong effects from both prosocial and antisocial peer norms (Van Hoorn, Van Dijk, Meuwese, et al., 2016) through social learning, suggesting that the process is similar, irrespective of the direction of influence. We hypothesize that adolescents with ADHD are more sensitive to feedback from the peer group, because they experience more peer rejection and difficulties in managing peer relations (Bagwell et al., 2001; Ferguson, 2000), which may lead to more conformity to be accepted by peers and perhaps gain status.

We exploratively test whether symptoms of inattention and hyperactivity‐impulsivity across the total sample are associated with sensitivity to impulsive and non‐impulsive peer feedback. While most of the existing ADHD literature relies on classified diagnostic groups, recent work also adopted a more dimensional approach to ADHD, supported by taxonomic and genetic studies (Coghill & Sonuga‐Barke, 2012; Nikolas & Burt, 2010). Also, there is some evidence that social functioning deficits vary by ADHD presentation (Solanto et al., 2009). Taken together, these findings suggest a more continuous relationship and a differential contribution of inattention and hyperactivity‐impulsivity symptoms to peer influence susceptibility.

## METHODS

### Participants

The study and its main analyses were preregistered and can be retrieved from the Open Science Framework at https://osf.io/mz3d2/. We have conducted this work in accordance with the preregistration and explicitly indicate throughout the manuscript if we deviate from the preregistration. The sample consisted of early to late adolescent males, ages 13–23, with (*n* = 51) and without (*n* = 62) ADHD. Data from one additional participant was missing due to technical issues. Participants were primarily recruited through previous studies that targeted both clinical and typically developing samples (Dekkers et al., 2019; Ma et al., 2020), and additionally through a recruitment website.

Participants with ADHD were included if they met the following criteria: (a) previous (lifetime) ADHD diagnosis by a licensed psychologist or psychiatrist, and (b) a current ADHD classification (all presentations) based on a parent/caretaker interview with the ADHD section of the Diagnostic Interview Schedule (DISC‐IV; Shaffer et al., 2000) conducted at their home. The DISC‐IV was adjusted to reflect changes from DSM‐IV to DSM‐5 where necessary. A total of 12 participants with ADHD recruited from a previous study comparing youth with and without ADHD (Dekkers et al., 2019), were in partial remission for a current ADHD classification based on the DISC‐IV. We maintained these participants in the current analyses.^2^ Comorbid disorders were not assessed with the DISC‐IV, but reported by parents.

Comorbid disorders were allowed and reported for *n* = 19 participants in the ADHD group (36% of the sample). Comorbid disorders included dyslexia (18%), dyscalculia (2%), oppositional defiant disorder (2%), depression (2%), and autism spectrum disorders, either as single comorbid diagnosis or co‐occurring with additional comorbid disorders (14%; this included dyslexia, dyscalculia, developmental coordination disorder, dysthymia, attachment disorder, and obsessive‐compulsive disorder). Note that percentages do not add up exactly due to rounding. Dyslexia was only allowed if parents reported that their child was able to read short sentences within 5 s.

The majority of the ADHD group (*n* = 30; 57%) was taking stimulant medication at the time of the study (53% methylphenidate; 4% dexamphetamines). Participants using methylphenidate discontinued medication 24 h before testing to reach total wash‐out (Greenhill & Ford, 2002). For participants using dexamphetamine, the required wash‐out period was 48 h (Wong & Stevens, 2012). Adolescents using atomoxetine, clonidine or antipsychotic medication were excluded. Participants without ADHD were included when parents reported no current psychiatric diagnoses for their child. Dyslexia/dyscalculia was allowed, and dyslexia occurred in *n* = 10 participants (16%).

Descriptive characteristics of the ADHD and typically developing (TD) group are presented in Table 1, for additional information see Supporting Information S1. We further describe our sample using parent report on the Child Behavior Checklist (CBCL; Achenbach et al., 2001) to assess emotional and behavioral problems, the Disruptive Behavior Disorder Rating Scale (DBDRS; Oosterlaan et al., 2000) to examine ADHD symptoms and potential comorbid behavioral disorders (ODD and CD), and Social Responsiveness Scale‐2 (SRS‐2; Roeyers Thys, Druart, De Schryver, & Schittekatte, 2011) to assess for symptoms in the autism spectrum. To test for possible confounding group differences, we also acquired estimated IQ scores for intelligence (see Supporting Information S1). The estimated IQ scores fell within the average to above average range for all participants and did not differ between the ADHD group and TD group.

**TABLE 1:** Descriptive characteristics of the attention‐deficit/hyperactivity disorder and typically developing group

Mean (SD), Range	TD	ADHD	Stats
	(*N* = 62)	(*N* = 51)	
Age	17.81 (2.23)	17.35 (2.30)	*t*(111) = 1.07, *p* = .288
	13.62–23.43	13.20–22.93	
WISC/WAIS estimated IQ	106.61 (12.10)	107.62 (13.75)	*t*(111) = −0.41, *p* = .680
	80–125	80–138	
Parental income	Low: 2%	Low: 10%	*X* ^2^(2101) = 4.20, *p* = .122
	Middle: 10%	Middle: 10%	
	Upper: 81%	Upper: 67%	
	Missing: 7%	Missing: 13%	
DISC ADHD presentation (C/I/HI/partial remission)	–	11/28/0/12	*–*
DBDRS inattention	3.75 (4.11)	14.41 (6.02)	*t*(108) = −11.00, *p* < .001
	0–17	2–26	
DBDRS hyperactivity‐impulsivity	1.52 (2.34)	8.48 (5.74)	*t*(109) = −8.63, *p* < .001
	0‐11	0‐22	
DBDRS ODD	2.05 (2.70)	5.00 (4.98)	*t*(109) = −3.98, *p* < .001
	0‐12	0‐22	
DBDRS CD	0.30 (0.76)	0.82 (1.34)	*t*(109) = −2.60, *p* = .011
	0–4	0–6	
CBCL impairment in general functioning	15.60 (16.52)	43.92 (21.98)	*t*(108) = −7.71, *p* < .001
	0–85	8–107	
SRS‐2 autism traits	25.27 (14.10)	42.32 (21.15)	*t*(108) = −5.05, *p* < .001
	8–81	13–105	

*Note*: Income was parent‐reported and classified as lower range for gross family yearly income <€31.000, middle range between €31.000‐€41.000, and upper range for >€41.000 (Central Bureau of Statistics, 2014).

Abbreviations: ADHD presentation C, combined; CBCL, Child Behavior Checklist; DBDRS, Disruptive Behavioral Disorders Rating Scale; DISC, Diagnostic Interview Schedule for Children (DISC‐IV); HI, hyperactive/impulsive; I, inattentive; SRS‐2, Social Responsiveness Scale‐2; WAIS, Wechsler Adult Intelligence Scale (WAIS‐III); WISC, Wechsler Intelligence Scale for Children (WISC‐V‐NL).

### Measures

#### Peers delay discounting task

We adapted a standard delay discounting paradigm in which the amount of the immediate reward and delay duration are varied in order to obtain a discounting function (de Water et al., 2014). The discounting task involved 24 choices between a smaller reward that was available immediately, and a larger reward that was available after a delay. The amount of the immediate reward was varied based on participants' choices (Du et al., 2002), and varied between €0.15 and €9.85. The delayed reward was fixed at €10. The delay preceding the larger reward varied between 2, 14, 30 and 90 days. Six unique choices were presented for each of the four delays. For example, the first choice was always between €5 today and €10 after a delay (e.g. €5 today or €10 after 2 days). If the participant chose the immediate reward, its amount would be decreased by half on the next trial (e.g., €7.50 today or €10 in 2 days). If the delayed reward was chosen, the value of the immediate reward would be decreased by half on the next trial (e.g., €2.50 today or €10 in 2 days). The subjective value of the delayed reward for each delay was defined as the hypothetical value of the immediate reward on a seventh trial at that delay (Du et al., 2002). These subjective values were used to compute the area under the curve (AUC), which ranges between 0 and 1, in which smaller values indicate an increased preference for immediate rewards (Myerson et al., 2001).

The task can be designated as potentially real, because participants were informed that one round would be selected as a reward at the end of the task, and each choice was therefore assumed to have been made as if it had real‐life consequences (Scheres, de Water et al., 2013). To make the rewards more explicit, participants were presented with real money on the table during the task: one €0.50 coin, one €1 coin, one €2 coin, one €5, and one €10 bill. The €10 bill was related to the largest amount of money that was included in the choices in the task.

**FIGURE 1:**
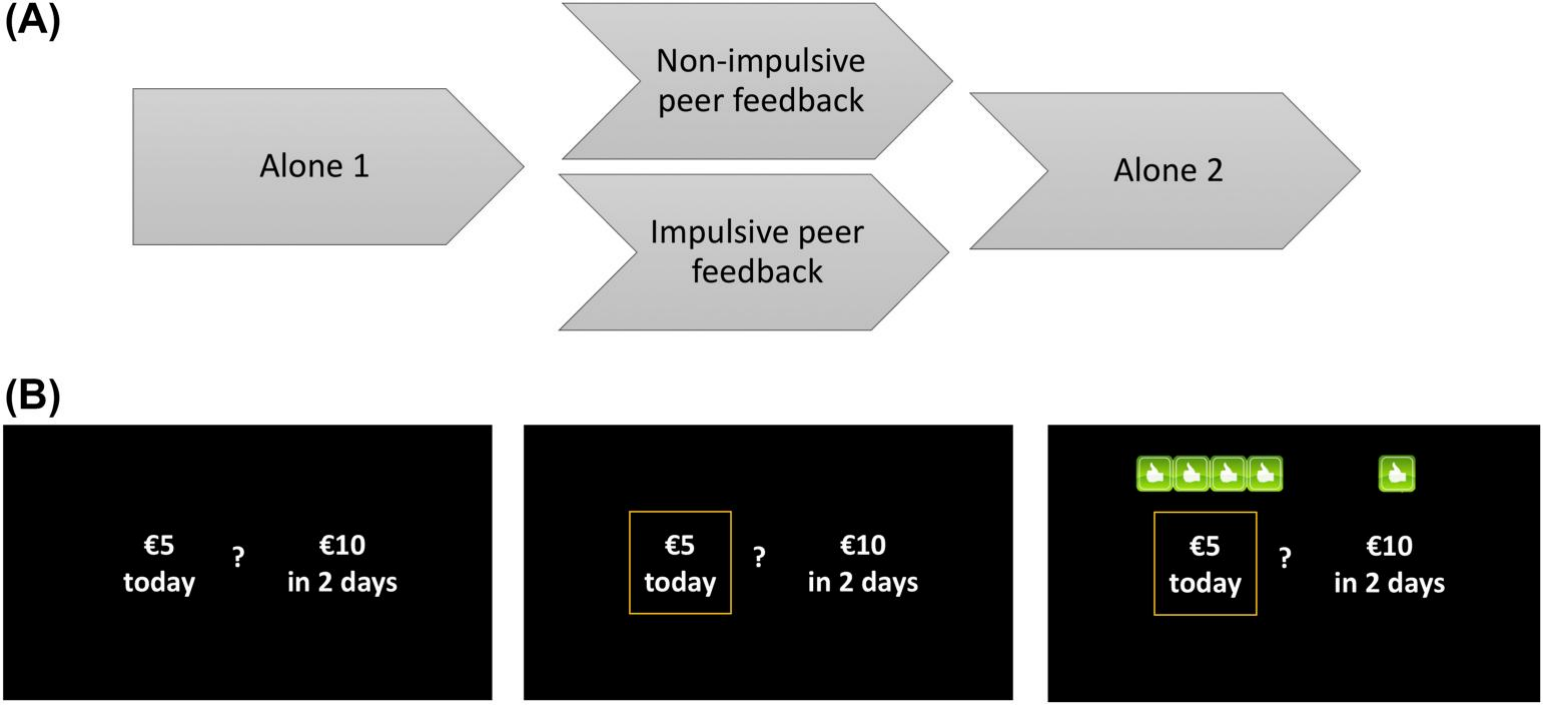
Delay discounting task with a between‐subjects peer influence condition. Panel A: task design. Panel B: screens indicating the two options, the choice made indicated by the yellow square, and an example of subsequent impulsive peer feedback. In this case, four peers gave a thumbs up for the impulsive choice, and one peer gave a thumb up for the non‐impulsive choice

The delay discounting task consisted of three fixed‐order within‐subject conditions: alone, with alleged peer feedback and alone again (cf. Van Hoorn et al., 2016). We utilized a between‐subjects design in which we compared two types of feedback: impulsive peer feedback and non‐impulsive peer feedback (Figure 1A). Peer feedback consisted of images of thumbs up or ‘likes’ of five fictitious peers. The thumbs up were presented on the screen (in fixed order, the same for each participant) after the participants made their decision. In the impulsive peer feedback condition, the majority of peers liked the impulsive option (i.e., 3, 4, or 5 fictitious peers gave a thumbs up, averaging to 75% of the five thumbs up across trials, for the smaller sooner reward). The task was programmed such that following the majority of impulsive peers' likes corresponded with an AUC of 0.018 (impulsive). In the non‐impulsive peer feedback condition, the majority of peers liked the non‐impulsive option, that is, the larger, delayed reward (see Figure 1B). Following the majority of peers' likes in the non‐impulsive condition corresponded with an AUC of 0.993 (patient).

#### Peer influence manipulation

Peer feedback came from five same‐sex, same‐age peers who were supposedly participating at another location. All participants saw the same peers in the task. To increase credibility, participants were shown a pre‐recorded video of a Zoom meeting, with five peers who were muted and waiting for the study to start, as well as one empty screen to represent the participant's Zoom screen. An experimenter showed the video to the participants and said that the peers were ready to get started, and that the laptop would be connected with their computers to play the task together. The peers were actors and friends of actors recruited through the peer actor database from a previous study (see van Hoorn, Van Dijk, Guroglu et al., 2016), with current ages between 12 and 19 years. They received a one‐time endowment of €5 to record the Zoom meeting at the university building and have their picture taken for the task, to which they consented beforehand. We used a screenshot from the Zoom meeting during the task, in which we asked the actors to show a neutral facial expression (see Supporting Information S1 for more information on quality checks for the social manipulation).

### Procedure

This task was administered as part of a larger study that was approved by the medical ethical committee of the Leiden University Medical and the ethical committee of the Institute of Psychology at Leiden University. Parents signed an informed consent form prior to completing the online parent questionnaires, and a separate informed consent to participate in the DISC‐interview (ADHD group only). When at the lab, all participants and their parents (in case of minors) signed an informed consent form prior to the start of the study. All participants were tested one‐on‐one by trained experimenters, who could provide help when necessary. Following the task instructions, including six practice trials, participants were informed that the computer would randomly pick one trial that would be selected for pay‐out. In fact, all participants received €2.50 as bonus compensation for this task, €40 for participating in the larger study, as well as a goodie bag that contained small gifts. Parents received €10 to complete a set of online questionnaires, and €10 for participating in the DISC‐interview (the latter for the ADHD group only). The task was presented on a laptop using E‐prime (version 3), had self‐paced timing and lasted about 10 min on average.

### Statistical analyses

The dependent variable was the AUC in each of the three within‐subjects conditions: alone, with alleged peer feedback and alone again (cf. Van Hoorn, Van Dijk, et al., 2016). Our main preregistered analysis used a mixed ANOVA testing the between‐subjects effects of Feedback Type (Impulsive/Non‐impulsive) and Group (ADHD/TD) on the change in individual's AUC towards peer feedback and when playing alone again (Alone 1/with feedback/Alone 2), with repeated measures of the last factor and age as a covariate. Since previous work also emphasizes a more dimensional approach to ADHD (Coghill & Sonuga‐Barke, 2012; Nikolas & Burt, 2010), we exploratively test a more continuous relationship between inattention and hyperactivity–impulsivity symptoms and susceptibility to social influence. We conducted exploratory regression analyses with the difference score AUC in Alone 2—AUC in Alone 1 as the dependent variable, separately for each Feedback condition (impulsive/non‐impulsive). Age and scores on DBDRS Inattention and Hyperactivity–Impulsivity (mean‐centered) were included as independent variables in these analyses.^3^

## RESULTS

### Non‐impulsive peer feedback decreases impulsivity

**FIGURE 2:**
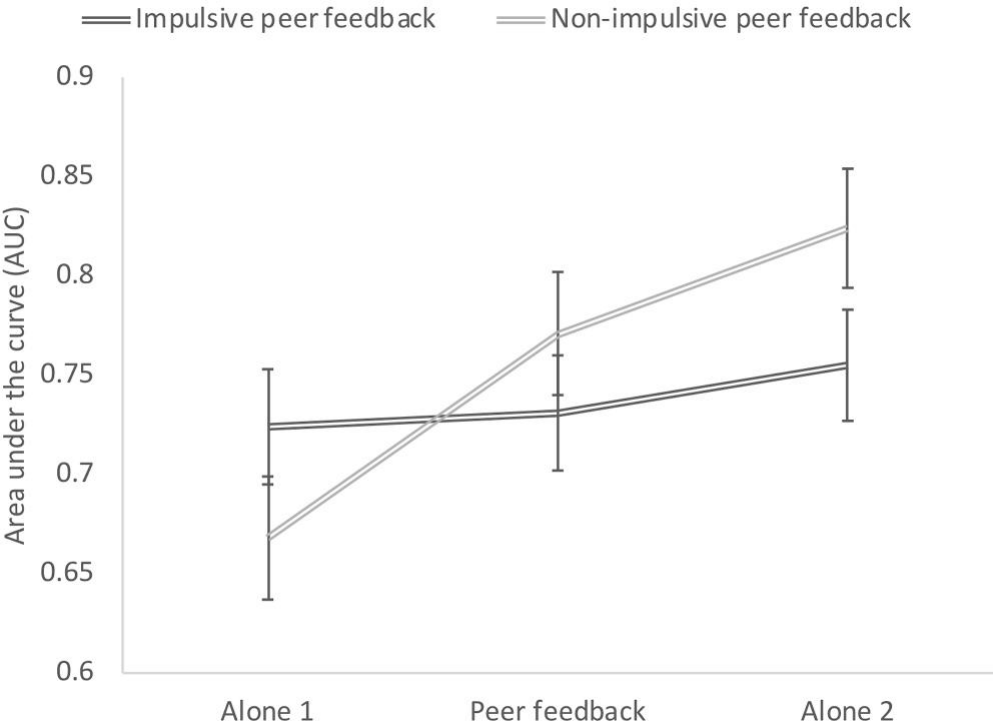
Effect of peer feedback on impulsive choice in the adapted delay discounting task. Error bars represent one standard error of the mean (SE)

Results from the mixed ANOVA indicated no main effects of Feedback type (*p* = .663) or Condition (*p* = .109), but did reveal an interaction of Feedback type by Condition, (*F*(2,216) = 14.610, *p* < .001, *partial η* ^*2*^ = 0.119). There was no main effect nor an interaction effect of Group (*ps* > .2), indicating that the effects of peer feedback on impulsive choice were similar for adolescents with and without ADHD. Finally, there was no effect of the covariate Age (*F*(1,108) = 1.812, *p* = .181). Means for the area under the curve (AUC) as a measure for impulsive choice in each condition are displayed in Figure 2.

Bonferroni‐adjusted post‐hoc tests were used to unpack the interaction between Feedback type and Condition. In the impulsive peer feedback condition, participants' decision‐making was similar when they were alone, during peer feedback, and alone again (*p*s > .2), suggesting no effects of impulsive peer feedback. In contrast, results yield a significant decrease in impulsive choice in the non‐impulsive peer feedback condition, such that the AUC in Alone 1 < Peer feedback (*p* < .001), Peer Feedback < Alone 2 (*p* = .002), and Alone 1 < Alone 2 (*p* < .001; see Figure 2). Hence, the effects of peer feedback remain and appear to become even stronger when adolescents go back to making decisions alone.

### Inattention and hyperactivity–impulsivity relate to peer feedback susceptibility

**FIGURE 3:**
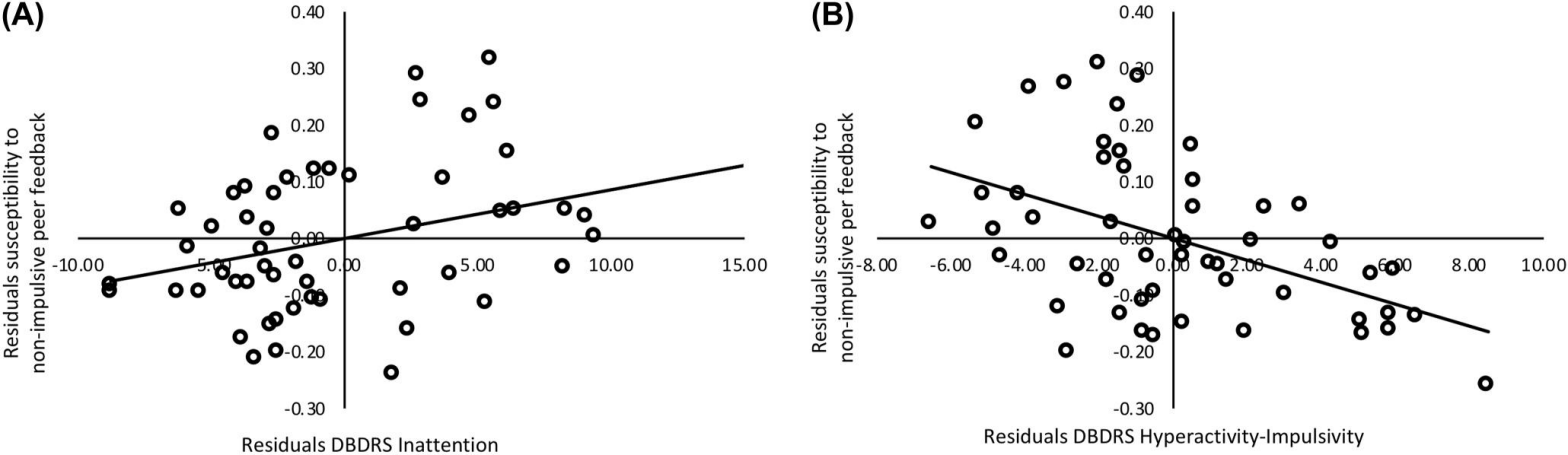
Partial regression plots between symptoms of inattention (panel A) and hyperactivity‐impulsivity (panel B) and their relation with susceptibility to non‐impulsive peer feedback across the total sample. Partial regression plots are displayed to show the relation of inattention and hyperactivity‐impulsivity symptoms controlling for the other independent variables in the model

Given the differential effects on peer influence across feedback condition, exploratory regression analyses were run separately for the Impulsive and Non‐impulsive feedback conditions. In the Impulsive feedback condition, susceptibility to peer feedback was not predicted by individual differences in hyperactivity or inattentive symptoms (*p* = .893). In the Non‐impulsive feedback condition, the regression model was significant (*F*(3,52) = 5.925, *R* ^2^_Adj_ = 0.221, *p* = .002). Inattention symptoms were a positive predictor, (*β* = .453, *t* = 2.50, *p* = .016), while hyperactivity–impulsivity (*β* = −.712, *t* = −3.80, *p* < .001) and age (*β* = −.365, *t* = −2.83, *p* = .007) were negative predictors. This demonstrates that in the total sample, more inattention symptoms related to greater susceptibility to non‐impulsive peer feedback (i.e., becoming less impulsive), whereas being older and having more hyperactivity‐impulsivity is associated with reduced susceptibility (see Figure 3).

## DISCUSSION

The goal of this preregistered study was to examine peer feedback on impulsive choice in adolescent males (ages 13–23 years) with and without ADHD, distinguishing between impulsive and non‐impulsive peer feedback. The key finding was that non‐impulsive peer feedback decreased impulsive choice in both groups. The effect of non‐impulsive peer feedback sustained even after peers were no longer present (i.e., a carry‐over effect). In contrast, impulsive peer feedback did not alter impulsive decision‐making. We also explored individual differences in susceptibility to peer feedback and found that, irrespective of diagnosis, more inattention symptoms related to increased susceptibility to non‐impulsive peer feedback, whereas more hyperactivity‐impulsivity symptoms and being older were related to reduced susceptibility to non‐impulsive peer feedback. Together, these findings support the view that peers may provide an opportunity to change impulsive decision‐making for the better and emphasize individual differences in susceptibility to peer feedback related to inattention and hyperactivity‐impulsivity symptoms.

### Peer feedback decreases impulsive choice in adolescents with and without ADHD

A large body of research indicates that the peer context plays a major role in shaping adolescent decision‐making (Andrews et al., 2021). More specifically for impulsivity, peer influence studies using the delay discounting task have revealed that presence or observation of impulsive peers increase impulsive choice in adolescents (Gilman et al., 2014; O’Brien et al., 2011; Weigard et al., 2014). Here, we observed that non‐impulsive peer feedback decreased impulsive choice, although impulsive peer‐feedback did not increase impulsive choice. While the direction of our effects appears in contrast with earlier work, this inconsistency can be explained using social learning theory (Akers, 2011; Bandura, 2001). An important element in this theory is that peer influence works through internalizing social norms from the peer context. Yet, the peer influence process is active; adolescents do not just passively absorb the social norms from peers around them (Brechwald & Prinstein, 2011). This is apparent in our data since adolescents did not follow their peers' social norms in the impulsive peer feedback condition. Our participants' own norms (i.e., baseline delay discounting decisions) were quite different from the social norms relayed in the impulsive peer feedback condition, but relatively similar to peers' non‐impulsive social norms. In sum, our findings align with the idea that adolescents appear to be influenced by peers when social norms are relatively closely aligned with their own norms (Ciranka & van den Bos, 2019), and they do not blindly follow their peers' feedback.

We also observed a transfer of non‐impulsive peer feedback to subsequent individual decisions. Similar to previous results in the domain of prosocial behavior (Van Hoorn, Van Dijk, Meuwese et al., 2016), this carry‐over effect suggests that the social norms provided by the peer group are to some extent maintained over time. Interestingly, adolescents preferred larger delayed rewards even more in the subsequent alone round than during peer feedback. Speculatively, this may suggest that the social norms were not only learned but perhaps also internalized (Staub, 1972), due to confirmation in their preference for larger delays. Future studies could include a follow‐up after a few months to assess whether peer feedback continues to guide impulsive decisions over time, or employ a longitudinal design to get a better understanding of causality.

On a group level, we found that adolescents with and without ADHD were equally susceptible to peer influence from a group of five peers. Consistent with social learning theory (Akers, 2011; Bandura, 2001) and accounts of differential reward sensitivity (Sonuga‐Barke, 2002, 2003) we had expected to find that adolescents with ADHD would be *more* susceptible to peer influence. Alternatively, one might hypothesize that ADHD‐related executive function impairments, as well as positive illusory biases would yield *less* sensitivity to peer feedback. That is, adolescents with ADHD would be less likely to inhibit pre‐potent responses and consequently respond before considering contingencies and feedback from peers; are less likely to evaluate and consider possible contingencies and feedback from peers due to impaired working‐memory (Kofler et al., 2011; Patros et al., 2015), and more likely to overestimate their performance and feedback from peers due to positive illusory biases (Owens et al., 2007). While neither of these hypotheses were supported by the data, our findings resonate with a related study that also found no differences in susceptibility related to ADHD status when a single peer encouraged risk‐taking behavior (Dekkers et al., 2020). Taken together, the findings suggest that in principle adolescents with ADHD appear equally susceptible to peer influence, either from a single peer or a small group of peers. Note however that adolescents with ADHD affiliate with deviant peers more often (Nijmeijer et al., 2008), and therefore it has been suggested that they are less likely than typically developing peers to experience positive peer influence that could help them make less impulsive choices (Dekkers et al., 2020).

### Inattention and hyperactivity–impulsivity symptoms associated with susceptibility to peer feedback

While our main analysis did not show any group‐level differences related to an ADHD diagnosis, our exploratory findings revealed that variability in inattention, hyperactivity–impulsivity (Coghill & Sonuga‐Barke, 2012; Nikolas & Burt, 2010) and age were associated with adolescents' susceptibility to non‐impulsive peer feedback. Specifically, higher inattention was associated with greater susceptibility, whereas more hyperactivity–impulsivity symptoms and being older was related to reduced susceptibility. The age effect resonates with previous work that illustrates how the impact of social influence slowly decreases across age (Andrews et al., 2021; Knoll et al., 2015). To our knowledge, the current work is the first to examine how inattention and hyperactivity–impulsivity symptoms are associated with susceptibility to peer influence, especially related to the opportunities of non‐impulsive peer influence (but see Scheres et al., 2010 for a similar analytical approach with a delay discounting task).

Some studies have examined social difficulties associated with the behavioral phenotypes of each ADHD presentation (i.e., predominantly inattentive, hyperactive‐impulsive, or combined). The nature of social difficulties tends to vary between the different ADHD presentations, but it is clear that all presentations experience impairments in social decision‐making (Humphreys et al., 2016; Maedgen & Carlson, 2000), leading to difficulties with peer relations (Bagwell et al., 2001). On a symptom‐level, our results show that more inattention is related to greater susceptibility to peer influence. Consistent with previous work, this may fit with a greater lack of assertiveness (Solanto et al., 2009) or as a means to be accepted in a group and gain status (Brechwald & Prinstein, 2011) in individuals with the predominantly inattentive presentation of ADHD. On the other hand, greater hyperactivity‐impulsivity symptoms are related to lower susceptibility to non‐impulsive peer influence, perhaps due to lower (social) self‐control or working memory (Kofler et al., 2011; Patros et al., 2015; Solanto et al., 2009), which would leave less room for peer feedback to affect decision‐making.

Together, these exploratory findings may provide an explanation for the absence of group‐level (interaction) effects of ADHD in our main analyses on the effects of non‐impulsive feedback, given that inattention and hyperactivity–impulsivity symptoms had effects in the opposite direction. Moreover, ADHD is a very heterogeneous disorder, such that many different combinations of inattention and hyperactivity–impulsivity symptoms can lead to the diagnosis in all its presentations (Faraone et al., 2021). A continuous approach combined with disentangling contributions of different symptoms may be informative to investigate complex social processes such as peer influence. It is important to emphasize that the differential relations of ADHD symptoms with peer influence were only observed when adolescents were presented with non‐impulsive peer norms. Future research should further examine whether impulsive peer‐influence relates to inattention and hyperactivity–impulsivity symptoms. Potentially, when impulsive peer‐influence aligns more closely to an individual's own level of impulsivity, peer influence may be more symmetrical, swaying others towards more impulsive choice behavior (Ciranka & van den Bos, 2019). Moreover, it is an open question how our findings translate to other relevant domains of behavior in adolescents with ADHD such as risk‐taking and (pro)social behavior. Gaining more insights into susceptibility to peer influence in a wide range of domains may help identifying those adolescents who may particularly profit from positive peer influence, or those who are at risk for negative peer influence.

### Limitations and future directions

A few limitations of this study should be noted. First, we included a peer manipulation with optimal experimental control (i.e., peer feedback from five unknown peers). While the ages of these peers were not revealed to the participants, the peers' ages were not always perfectly matched, especially for our older participants. It is also unclear whether the effects would be similar if feedback came from adolescents' actual friends. Social identity theory (Islam, 2014) predicts that the effects would likely be stronger, given that adolescents more strongly identify with their friends than unknown peers. In that light, the current study represents a conservative test of the power of peers, which already showed promising findings.

Second, we did not examine the effects of our peer manipulation on (impulsive) behavior outside the experimental task. However, previous work has already shown that delay discounting task manipulations are related to health‐risk behaviors (e.g., smoking, consuming unhealthy foods; Amlung et al., 2016; Audrain‐McGovern et al., 2009; Jackson & MacKillop, 2016). It would be a valuable addition for future research to link the current experimental findings to real‐world impulsive or risk behaviors.

The third limitation that can be noted is that we only included males in the current study. Although ADHD is more prevalent in males (6%) than females (3%) before the age of 18 (Dalsgaard et al., 2020), future research could include females and examine whether they similarly benefit from non‐impulsive peer feedback. Given that girls generally show lower levels of hyperactivity–impulsivity (Gershon & Gershon, 2002), one hypothesis based on the current work could be that they are more susceptible to non‐impulsive peer influences. Future work employing a between‐subjects design should include a larger sample size for optimal power, including boys and girls, to replicate the current findings.

Finally, steep discounting of delayed rewards in delay discounting tasks has been described as a trans‐diagnostic mechanism (Amlung et al., 2019), as it is not only characteristic of adolescents with ADHD (Jackson & MacKillop, 2016; Marx et al., 2021; Patros et al., 2016), but also frequently observed in adolescents with disorders that are highly comorbid with ADHD, such as substance abuse (Reynolds & Fields, 2012) and conduct disorder (White et al., 2014). While examining the effects of comorbidity was outside of the scope of the current study, a broad clinical assessment targeting comorbidity would be an interesting avenue for future research, as interventions targeting delay discounting could potentially reduce not only ADHD symptoms, but also symptoms of comorbid disorders.

### Conclusions and implications for clinical practice

In conclusion, our study provides empirical support for the opportunities of positive peer feedback to decrease impulsive choice in adolescents with and without ADHD. Moreover, we exploratively showed that across our sample of adolescents there is substantial variability in susceptibility to peer feedback as a function of both hyperactivity‐impulsivity and inattention. Our findings suggest that it would be relevant to include a social component in an intervention to reduce impulsive choice in youth with ADHD (e.g., peer role models or peer feedback on behavior). This is in line with a recent review which indicates that behavioral peer interventions for ADHD are well‐established psycho‐social interventions (Evans et al., 2018). Interestingly, these programs mostly rely on trained staff or parents to facilitate successful behavior. Our findings suggest that involving (virtual) peer processes could be utilized in a similar way to decrease impulsive behaviors in adolescents with ADHD.

## CONFLICT OF INTEREST

The authors have declared that they have no competing or potential conflicts of interest.

## ETHICAL CONSIDERATION

This task was administered as part of a larger study that was approved by the medical ethical committee of the Leiden University Medical and the ethical committee of the Institute of Psychology at Leiden University.

## AUTHOR CONTRIBUTIONS

Jorien van Hoorn contributed to conceptualization, data curation, formal analysis, investigation, methodology, project administration, visualization, writing and editing. Erik de Water contributed to methodology, software, writing and editing. Tycho Dekkers contributed to conceptualization, methodology, writing and editing. Yehuda Pollak, Arne Popma, Brenda Jansen and Hilde Huizenga contributed to conceptualization, writing and editing. Anna van Duijvenvoorde contributed to conceptualization, formal analysis, funding aqcuisition, investigation, methodology, resources, supervision, writing and editing.

## Supporting information

Supporting Information S1

## Data Availability

The data that support the findings of this study are available from the corresponding author upon reasonable request.

## ENDNOTES

^1^
Practical considerations included reliance on hormone measures, unrelated to the current study.

^2^
As preregistered, we examine the effects of current versus past diagnosis and comorbidity. All analyses that included group comparisons were conducted with and without these participants, which did not affect any of the presented conclusions. Therefore, all participants were kept in the analyses reported in the results.

^3^
In Supporting Information S1, we report additional exploratory analyses, which were similar to the ones reported in the manuscript, but with a *relative* social influence variable as the dependent variable [also see Molleman et al. (2021)]. This allowed us to assess how much the AUC changed, relative to how much participants could have changed depending on their starting point (Alone 1) towards the AUC from peer feedback.
