# Supplementary material for: Peer feedback decreases impulsive choice in adolescents with and without attention‐deficit/hyperactivity disorder
Source: JCPP Adv. 2022 Feb 25;2(1):e12065. doi: 10.1002/jcv2.12065 (PMC10242953; doi:10.1002/jcv2.12065)
Supplement: Supplementary file 1 — Supporting Information S1 [file JCV2-2-e12065-s001.docx]

**Supporting Information**

**Appendix S1.**

**Supplementary Methods**

*Participants*

*Age distribution.* Figure S1 illustrates the age distribution of our sample, for ADHD and control group separately, showing that the majority of our participants was between 13 and 19 years old.

*Figure S1.* Age distribution of the sample.

*Missing descriptives data.* For one participant, a specific language impairment disorder was discovered upon arrival. This participant did not complete the questionnaires, but did complete the behavioral task with assistance and was kept in the sample. Full DBDRS scores were missing for 2 participants (1 ADHD; 1 TD), resulting in 62 TD and 49 ADHD scores. DBDRS inattention scores were missing for one additional participant, resulting in 48 DBDRS inattention scores. CBCL and SRS-2 data were missing for 3 participants (1 ADHD; 2 TD). Finally, missing data for parental income as reported in Table 1 were due to parents not knowing their income or not wanting to share their income.

*Intelligence.* We administered the subscale Similarities and Block Patterns from the Wechsler Intelligence Scale for Children (Wechsler, 2014; WISC-V-NL, participants age < 16;11) and Wechsler Intelligence Scale for Adults (Wechsler, 2008; WAIS-III, participants age 16;0+). For the majority of participants, there was estimated IQ information available from previous studies (n = 71 from Dekkers et al., 2019; n = 18 from Ma et al., 2020) which was obtained within the time span of 1 to 2 years before the start of the current study. Estimated IQ scores were obtained from the remaining 24 participants, resulting in IQ scores for the entire sample.

*Measures*

*Peer manipulation.* Participants rate the pictures of the peers after the task on estimated age as well as the dimensions neutrality (scale 1-7, not at all neutral to very much neutral), and likeability (scale 1-7, do not like at all to like very much). The estimated age of the actors fell within the age range of our participants (*M*_age_ (*SD*) = 16.32 (1.11), which indicates that these peers were valid for use within our paradigm. Participants rated the online peers with an overall neutrality of *M*(*SD*) = 4.69(0.89) and likeability of *M*(SD) = 4.90 (0.62), showing that they were perceived as sufficiently neutral and likable. A small number of participants questioned the social manipulation (*n* = 6). As pre-registered, all analyses were conducted with and without participants who questioned the social manipulation, which did not affect the results. Therefore, all participants were kept in the analyses reported in the results.

**Supplementary Results**

*Exploratory analyses with relative social influence*

We ran additional exploratory analyses with a relative social influence variable (see Molleman et al., 2021), that allowed us to assess how much the AUC changed, relative to how much participants could have changed towards the AUC from the observed peer feedback taking into account their initial level of impulsivity ((AUC Alone 2 – AUC Alone 1) / (AUC Peer Feedback – AUC Alone 1). We chose to use the difference between Alone 2 and Alone 1 in the numerator of the fraction because this provided an indication for sustained effects of peer feedback. This relative influence measure results in a positive score if adolescents moved towards the Peer Feedback AUC, negative score if they moved away from the Peer Feedback AUC, and (around) zero if their AUC remained the same over the course of the task. Note that if participant’s baseline impulsivity (Alone 1) is at the same level of the observed Peer’s AUC a relative social influence cannot be calculated. In our sample, four adolescents were therefore excluded from this analysis.

Across both between-subjects conditions the mean (SD) relative influence was 0.20 (0.43), range -0.62 to +1.00), suggesting that in general, adolescents tended to move towards the peer feedback AUC that they were presented with.

First, we examined whether adolescents with ADHD, compared to adolescents without ADHD were more susceptible to peer influence. A 2 (Group) × 2 (Feedback Type) univariate ANOVA on the susceptibility index with age as a covariate confirmed that adolescents conformed more to peers in the non-impulsive peer feedback condition (*M* = .521), while they were not influenced by impulsive peer feedback (*M* = -.070), as indicated by a main effect of Feedback type, *F*(1,104) = 91.517, *p* < .001, *partial η^2^* = .468). Similar to our previous analyses there was no main effect or interaction effect of Group (*p*s > .1), nor effects of the covariate Age (*F* (1,104) = 0.051, *p* =.821). These results strengthen the AUC findings from our first analysis by showing similar effects when we examine relative social influence.

Next, we explored whether individual differences in symptoms of inattention, hyperactivity and impulsivity (irrespective of group) would play a role in sensitivity to peer feedback. We ran a linear regression analysis on the relative social influence variable with symptom-level scores of inattention and hyperactivity-impulsivity (DBDRS; mean-centered), and Feedback type (non-impulsive coded as 0, impulsive as 1) as predictors. We included age as a covariate. All main effects and 2-way interactions with Feedback type were included. Our findings show main effects of Feedback Type (*t* = -9.667, *β* = -.662, *p* < .001), Inattention (*t* = 2.978, *β* = .434, *p* = .004) and Hyperactive-impulsive symptoms (*t* = -3.844. *β =* -.613, *p* < .001), which were particularly present in the non-impulsive feedback condition (Feedback Type × Inattention: *t* = -2,272, *β* = -.356, *p* = .025; Feedback Type × Hyperactivity-Impulsivity: *t* = 2.763, *β* = .460, *p* = .007). Age was not related to sensitivity to peer feedback (*p* = .366). As can be seen in Figure S2, more inattention symptoms were associated with increased sensitivity to non-impulsive feedback, whereas more hyperactivity-impulsivity symptoms. were associated with reduced sensitivity to non-impulsive peer feedback.

*
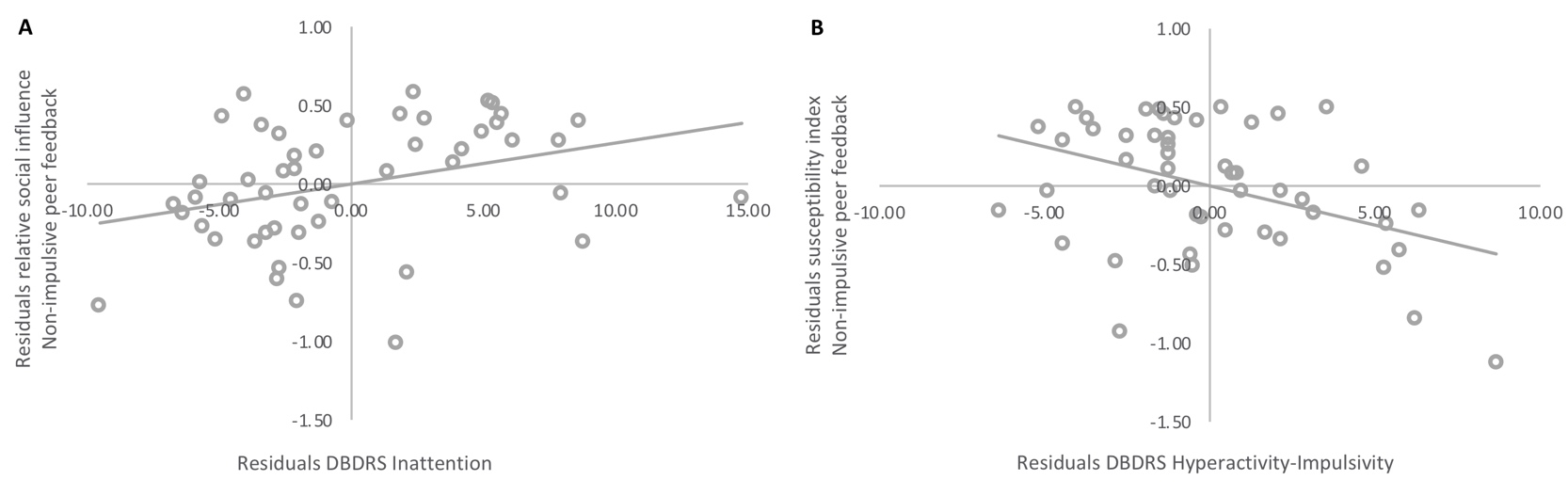
*

*Figure S2.* Partial regression plots between symptoms of inattention (panel A) and hyperactivity-impulsivity (panel B) and their relation with relative influence of non-impulsive peer feedback across the total sample. Partial regression plots are displayed to show the relation of inattention and hyperactivity-impulsivity symptoms controlling for the other independent variables in the model.
